# Retraction Statement: Circ‐TCF4.85 silencing inhibits cancer progression through microRNA‐486‐5p‐targeted inhibition of *ABCF2* in hepatocellular carcinoma

**DOI:** 10.1002/1878-0261.13451

**Published:** 2023-05-25

## Abstract

The above article, published online on January 10, 2020 in Wiley Online Library (wileyonlinelibrary.com), has been retracted by agreement between the journal Editor in Chief, Kevin Ryan and John Wiley and Sons Ltd.

The retraction has been agreed following an investigation into concerns raised by a third party, which revealed inappropriate duplications between this and two previously published studies [1, 2] by unrelated groups of authors. Thus, the editors consider the conclusions of this manuscript substantially compromised.

[1] MicroRNA‐126 inhibits tumor proliferation and angiogenesis of hepatocellular carcinoma by down‐regulating EGFL7 expression. DOI: 10.18632/oncotarget.11877 Oncotarget. 2016 Oct 11; 7(41): 66922–66934.

[2] ShRNA knock‐down of CXCR7 inhibits tumour invasion and metastasis in hepatocellular carcinoma after transcatheter arterial chemoembolization. DOI: 10.1111/jcmm.13119 J Cell Mol Med. 2017 Sep;21(9):1989–1999.

Reference

1 Gao J, Dai C, Yu X, Yin X‐B, Zhou F. Circ‐TCF4.85 silencing inhibits cancer progression through microRNA‐486‐5p‐targeted inhibition of ABCF2 in hepatocellular carcinoma. Mol Oncol. 2020;14:447–61. https://doi.org/10.1002/1878-0261.12603

The above article, published online on January 10, 2020 in Wiley Online Library (wileyonlinelibrary.com), has been retracted by agreement between the journal Editor in Chief, Kevin Ryan and John Wiley and Sons Ltd.

The retraction has been agreed following an investigation into concerns raised by a third party, which revealed inappropriate duplications between this and two previously published studies [1, 2] by unrelated groups of authors. Thus, the editors consider the conclusions of this manuscript substantially compromised.

[1] MicroRNA‐126 inhibits tumor proliferation and angiogenesis of hepatocellular carcinoma by down‐regulating EGFL7 expression. DOI: 10.18632/oncotarget.11877 Oncotarget. 2016 Oct 11; 7(41): 66922–66934.

[2] ShRNA knock‐down of CXCR7 inhibits tumour invasion and metastasis in hepatocellular carcinoma after transcatheter arterial chemoembolization. DOI: 10.1111/jcmm.13119 J Cell Mol Med. 2017 Sep;21(9):1989–1999.

Reference

1 Gao J, Dai C, Yu X, Yin X‐B, Zhou F. Circ‐TCF4.85 silencing inhibits cancer progression through microRNA‐486‐5p‐targeted inhibition of ABCF2 in hepatocellular carcinoma. Mol Oncol. 2020;14:447–61. https://doi.org/10.1002/1878-0261.12603

